# Supplementary material for: clotFoam: An Open-Source Framework to Simulate Blood Clot Formation Under Arterial Flow
Source: ArXiv. 2023 Aug 1:arXiv:2304.09180v3. Preprint. [Version 3] (PMC10153289)
Supplement: 1 [file NIHPP2304.09180V3-supplement-1.pdf]

Supporting Information

Mesh generation for 3D hemostasis example

To define the mesh in OpenFOAM, we utilize 3 blocks as seen in Figure E.8. The creation of holes in the vertical channels at the interface with the injury channel is accomplished using the `mergePatchPairs` function. The dimensions of the H-domain, which serve as the computational domain, are provided in Table E.11, following the description given in Schoeman et al. [14]. The mesh is defined so that the cells in the injury channel have a uniform spacing $\Delta x = \Delta y = \Delta z = 2.5\ \mu$m. The mesh outside of the injury channel is graded for computational efficiency. The resulting mesh, as shown in Figure E.8, consists of 37,248 finite volume cells. The specific grading parameters used to generate the mesh can be found in the file `tutorials/Hjunction3D/system/blockMeshDict` in the *clotFoam* repository on GitHub. These parameters were calculated using the free blockMesh grading tool [26].

Width of vertical channel (w_{chan})	100 μm
Height of vertical channel (h_{chan})	50 μm
Length of vertical channel (ℓ_{chan})	150 μm
Width of injury channel (w_{inj})	50 μm
Height of injury channel (h_{inj})	20 μm
Length of injury channel (ℓ_{inj})	150 μm

Table E.11: Dimensions of the computational domain of the H-shaped microfluidic device.

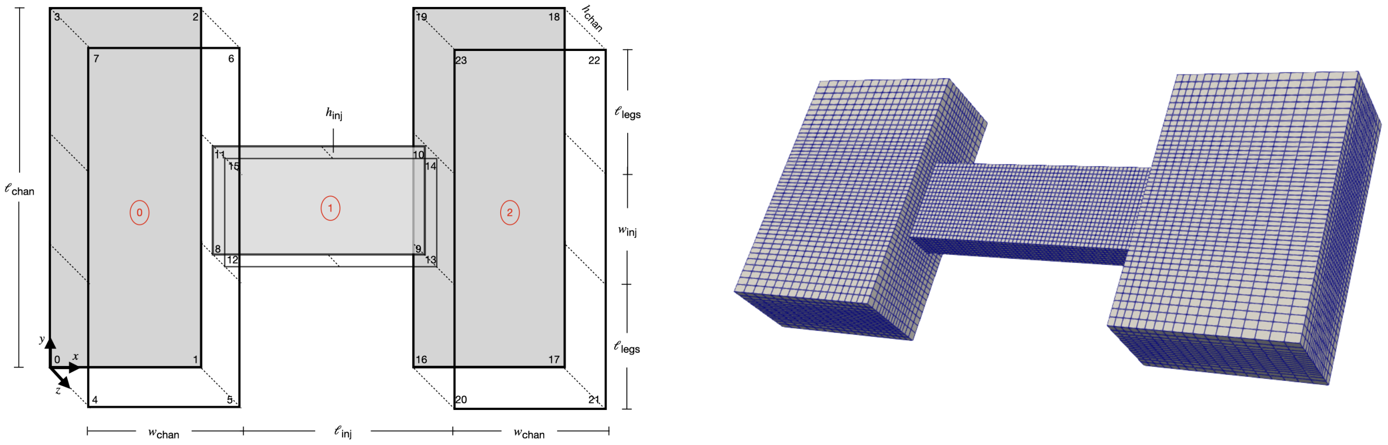

Figure E.8: Meshing the H-shaped microfluid device.

Mesh convergence study

To evaluate the accuracy and convergence of the *clotFoam* solver, we performed a mesh convergence study in a rectangular domain measuring 120 μm in length, 30 μm in height,

and with an injury length of 45 μm. The objective of this study was to assess the error by comparing the solution between a fine mesh and a coarse mesh. We utilized five different mesh discretizations, with uniform cell heights and widths (h) of 3, 1.5, 0.75, 0.375, and 0.1875 μm. In this study, the finest mesh was denoted as $h_{\text{fine}} = 0.1875$ μm, and its corresponding solution was represented by ψ_{fine}. To facilitate the comparison of solutions across different mesh resolutions, we employed the `Resample with Dataset` filter in ParaView to interpolate the coarse meshes onto the finest mesh. To ensure numerical stability, the time step was selected as a function of h in a way that the maximum Courant number (C_0) did not exceed 0.75 for each mesh. The Courant number is defined as $C_0 = \frac{\Delta t \|\vec{\mathbf{u}}\|}{h}$, where Δt represents the time step and $\|\vec{\mathbf{u}}\|$ is the magnitude of the velocity field.

The relative error for each solution ψ_{h_k} with mesh h_k is calculated using the following formula:

$$E(\psi_{h_k}) = \frac{\|\psi_{\text{fine}} - \psi_{h_k}\|_{\ell_2}}{\|\psi_{\text{fine}}\|_{\ell_2}}, \tag{E.1}$$

where the ℓ_2 norm is defined for a solution vector $\vec{\mathbf{x}} \in \mathbb{R}^n$ as:

$$\|\vec{\mathbf{x}}\|_{\ell_2} = \sqrt{\sum_{i=1}^{n} x_i^2}, \tag{E.2}$$

The convergence rate, denoted by r, was approximated by fitting a line to the error vectors on a log-log scale using least squares.

In Figure E.9, we present the errors at time $t = 5$ seconds for four different species that represent various components of the clotting model:

1. S_1 is a fluid-phase biochemical species that enters the domain with a fixed concentration and reacts on subendothelium with E_0 to produce the enzyme E_1.
2. E_1 is a fluid-phase species that enters the domain through a reactive boundary condition on the subendothelium.
3. $P^{m,u}$ is fluid-phase platelet species that enters the domain with a marginated profile and experiences hindered transport in the presence of a growing thrombus. This species has reaction terms that depend on the adhesion region and biochemical agonists such as ADP and thrombin.
4. $E_2 + E_2^b$ represents the total thrombin concentration and serves as a surrogate measure of overall clot formation. E_2 is a fluid-phase species, while E_2^b is platelet-bound.

The error plot in Figure E.9 demonstrates that the fluid-phase biochemical species converge with second-order accuracy, while the platelet-related species exhibit a super-linear convergence rate.

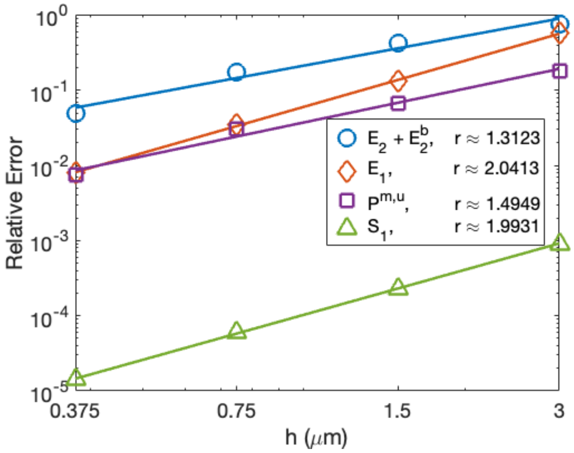

Figure E.9: Mesh convergence study in a rectangular domain. Errors are calculated at $t = 5$ seconds with respect to a fine mesh solution ψ_{fine} ($h = 0.1875$ μm), which serves as a reference solution. The approximate convergence rate r is displayed in the legend for each species.

Effect of mesh topology

To investigate the impact of mesh topology on thrombus formation, we generated an unstructured mesh using the open-source software Gmsh, as shown in Figure E.10. Our preliminary findings suggest that the specific mesh topology has a limited effect on the overall growth of the thrombus. However, we observed variations in clot density, with less dense regions occurring in areas where the mesh topology is more skewed. It is important to note that these observations are based on a single test with an unconventional mesh, and further investigations incorporating carefully selected discretization schemes may help mitigate this issue.

To assess the influence of mesh topology on thrombus growth, we conducted a comparative study using two different mesh configurations within a rectangular domain, as depicted in Figure E.10. The left column represents simulations conducted using a structured mesh, while the right column corresponds to simulations conducted using an unstructured mesh. Our comparison revealed similar patterns of thrombus growth in both simulations. We quantitatively evaluated the differences in thrombi by calculating the integral of the bound platelet fraction over the domain and normalizing it by its maximum value, resulting in the "normalized amount of bound platelets." The bottom plots in Figure E.10 demonstrate that the error in the normalized amount of bound platelets is less than 7% between the structured and unstructured mesh configurations after 600 seconds of simulation.

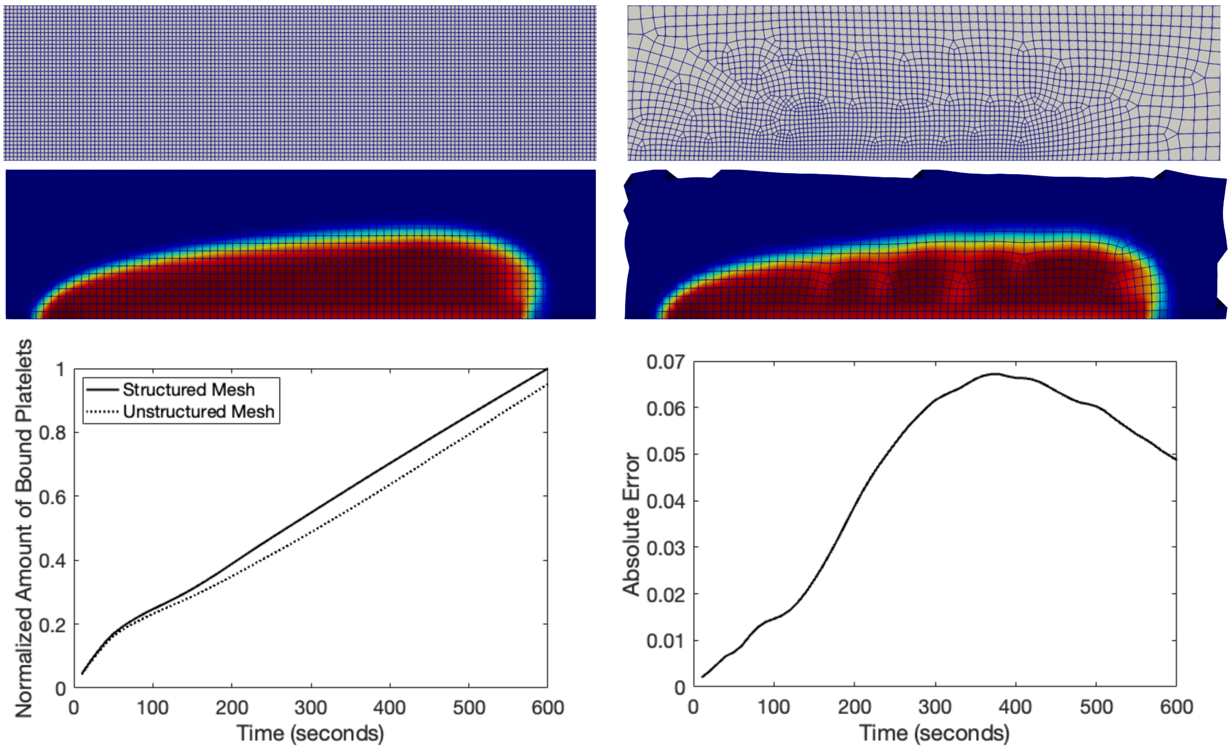

Figure E.10: Comparison of the effect of mesh topology on thrombus growth for a rectangular domain measuring 240 μm by 60 μm. The top row depicts a structured (uniform) mesh on the left and an unstructured mesh on the right. The second row provides a close-up view of the bound platelet fraction in a 120 μm long by 30 μm high region surrounding the thrombus after 600 s of simulation. The bottom row quantifies the discrepancy between the simulations by integrating the bound platelet fraction over the entire domain and normalizing it by the maximum value of that quantity.

Quantitative validation

To quantitatively validate the *clotFoam* solver, we conducted comparative studies with the works of Leiderman and Fogelson [3] and Schoeman et al. [14]. It is important to acknowledge that the clotting model implemented in *clotFoam* is a significantly reduced version of the Leiderman and Fogelson model. This reduction was intended to provide researchers with a flexible framework that can be adapted to suit their specific research needs and objectives. As a result, direct comparisons between these models can be challenging due to inherent differences in their formulations. However, considering the results obtained from these comparative studies, along with the mesh convergence analysis depicted in Figure E.9, it becomes evident that *clotFoam* demonstrates reliability in simulating thrombus growth under arterial flow conditions.

Comparison with Leiderman and Fogelson 2011

In the first comparative study, we compared thrombus growth from the *clotFoam* solver to Figure 8 in Leiderman and Fogelson [3]. We simulated thrombus growth under three shear rates (500, 1000, and 1500 s^{-1}) with four different inlet platelet profiles (P_0, P_1, P_2, P_3 as defined in Figure 3 of Leiderman and Fogelson) that have increasing margination ratios. To quantify the differences, we computed the 'area' in which the bound platelet concentration

exceeded 10%, 50%, and 90% of $P_{\max}$, respectively. The first percentage provides a measure of the overall thrombus size, while the latter two percentages provide information about the bound platelet density distribution within the thrombus. These 'areas' were computed in ParaView by counting the grid cells in which the various platelet concentration levels were exceeded, and are presented in Figure E.11.

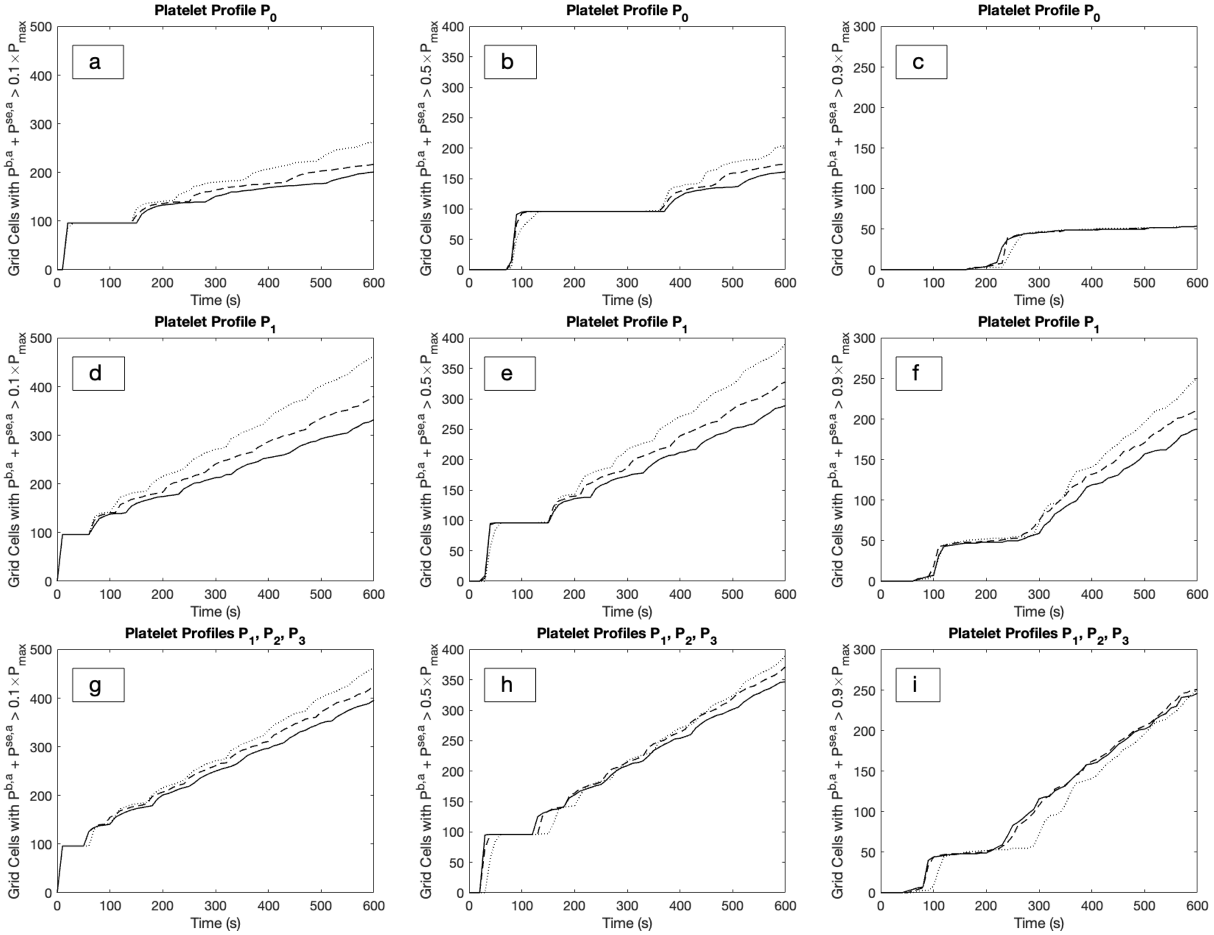

Figure E.11: Quantitative analysis of clot 'area' for comparison with Fig. 8 in Leiderman & Fogelson [3]. The top row (a-c) presents results obtained using a uniform platelet profile. The middle row (d-f) shows results obtained using a fixed nonuniform platelet profile P_1, as defined in Fig. 3 of Leiderman & Fogelson [3]. The bottom row (g–i) displays results obtained using platelet profiles P_1, P_2, and P_3 for wall shear rates of 500, 1000, and 1500 s^{-1}, respectively. In all cases, the dotted line, dashed line, and solid line represent wall shear rates of 500, 1000, and 1500 s^{-1}, respectively.

Similar to the plots in Leiderman and Fogelson [3], we observed the appearance of a temporary plateau at an area of 100 grid cells. This corresponds to the region where the adhesion rate function $k_{\text{adh}}H_{\text{adh}}(x)$ is non-zero. However, for the highest concentration of bound platelets, when the bound platelets concentration exceeded 90% of $P_{\max}$, we observed a plateau at an area of 50 grid cells. This indicates that only half of the adhesion region exceeds a density of 90% of $P_{\max}$. This behavior can be seen in Figure 5 of our manuscript, where the adhesion region at the bottom of the thrombus has a less dense concentration compared to the rest of the clot. Given the reduced nature of the *clotFoam* model, it is

not surprising that our results for the timing of plateaus, overall clot growth, and shear rate dynamics differ from those published by Leiderman and Fogelson. However, we do observe similar characteristics of thrombus growth, with the curves in Figure E.11 following the same general trajectories as those reported by Leiderman and Fogelson.

Comparsion with Schoeman et al. 2016

In the second comparative study, we conducted an assessment of thrombus formation over time by comparing our results with Figure 5 of Schoeman et al. [14]. Our primary objective with the illustrative example of hemostasis in a microfluidic device using the *clotFoam* software was to demonstrate its capability in simulating clot formation within such a device. However, it is important to acknowledge that the platelet model employed by *clotFoam* does not incorporate crucial factors like von Willebrand factor (vWF) or shear dependence in the platelet aggregation process, which are known to play significant roles in high shear settings, such as those encountered in the H-shaped microfluidic device described by Schoeman et al.

Despite the limitations of the platelet aggregation model in *clotFoam*, Figure E.12 illustrates characteristic platelet accumulation in the injury channel for whole blood on collagen and tissue factor (TF). The plot shows that as time progresses, the platelet accumulation increases until the injury channel reaches full occlusion at approximately 30 minutes, resulting in a plateau in the amount of bound platelets. This behavior is similar to what is observed in the Schoeman et al. paper, although the time scales differ. These findings demonstrate that the base clotting model in *clotFoam* is capable of simulating hemostasis in a microfluidic device. Researchers can easily modify the model to incorporate the effects of shear, von Willebrand factor (vWF), thromboxane A2, fibrin, and other relevant biochemical species involved in the coagulation process. The primary objective of developing *clotFoam* was to provide researchers with a versatile framework that can be easily customized and adapted to different clotting models. This flexibility allows for further exploration and customization to meet the specific research requirements of various studies in the field of hemostasis and thrombosis.

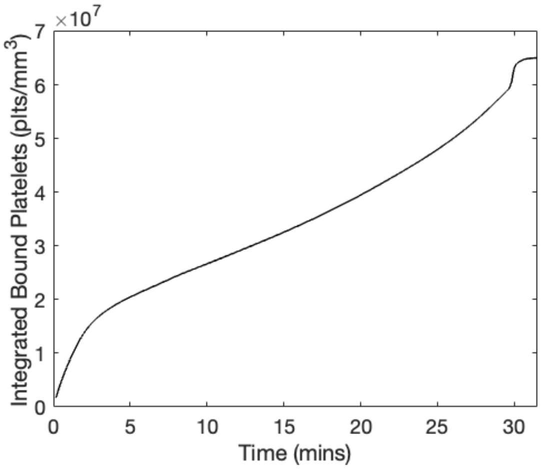

Figure E.12: Characteristic platelet accumulation in the injury channel for whole blood on collagen-TF. The plot shows the progressive accumulation of platelets over time until the injury channel reaches occlusion at approximately 30 minutes.
